# Jointly Embedding Protein Structures and Sequences through Residue Level Alignment

**DOI:** 10.1103/prxlife.2.043013

**Published:** 2024-11-19

**Authors:** Foster Birnbaum, Saachi Jain, Aleksander Madry, Amy E. Keating

**Affiliations:** Department of Biology, Massachusetts Institute of Technology, Cambridge, Massachusetts 02139, USA and Computational and Systems Biology, Massachusetts Institute of Technology, Cambridge, Massachusetts 02139, USA; Department of Electrical Engineering and Computer Science, Massachusetts Institute of Technology, Cambridge, Massachusetts 02139, USA; Department of Electrical Engineering and Computer Science, Massachusetts Institute of Technology, Cambridge, Massachusetts 02139, USA; Department of Biology, Massachusetts Institute of Technology, Cambridge, Massachusetts 02139, USA and Department of Biological Engineering, Massachusetts Institute of Technology, Cambridge, Massachusetts 02139, USA

## Abstract

The relationships between protein sequences, structures, and functions are determined by complex codes that scientists aim to decipher. While structures contain key information about proteins’ biochemical functions, they are often experimentally difficult to obtain. In contrast, protein sequences are abundant but are a step removed from function. In this paper, we propose residue level alignment (RLA)—a self-supervised objective for aligning sequence and structure embedding spaces. By situating sequence and structure encoders within the same latent space, RLA enriches the sequence encoder with spatial information. Moreover, our framework enables us to measure the similarity between a sequence and structure by comparing their RLA embeddings. We show how RLA similarity scores can be used for binder design by selecting true binders from sets of designed binders. RLA scores are informative even when they are calculated given only the backbone structure of the binder and no binder sequence information, which simulates the information available in many early-stage binder design libraries. RLA performs similarly to benchmark methods and is orders of magnitude faster, making it a valuable new screening tool for binder design pipelines.

## I. INTRODUCTION

An important goal in biology is to determine the relationships between protein sequence, structure, and function. Protein structures underlie biochemical functions such as binding and catalysis, but they are often expensive and time-consuming to resolve (e.g., via x-ray crystallography, NMR, or cryoelectron microscopy).

In contrast, the amino-acid sequences of proteins are far more accessible [1], but detailed information about function can be difficult to extract from sequence alone. There is thus considerable interest in matching the sequence of a protein to its structure using computational methods. Exciting progress has been made on problems that can be framed as sequence-structure alignment problems, such as structure prediction [2], sequence prediction [3,4], and binder design [5]. To solve such problems using machine learning, practitioners need to build encoders that informatively represent either the structure or the sequence.

Several groups have developed large language model encoders of protein sequences [6–8]. These models learn evolutionary patterns from sequence data and generate powerful representations of the underlying protein. However, although in theory protein sequences and structures contain the same information, in practice some tasks benefit enormously from an embedding of an input structure. For example, binder design is greatly facilitated by an encoding of a target structure that can properly condition the structure of a binder to match the specific, high-resolution structure of the target [5].

Structure encoders are limited by data availability. Whereas the UniProt database contains 60 million protein sequences [1], the Protein Data Bank (PDB)—the most comprehensive public database of protein structures—contains only 220 000 structures (as of June 2024), many of which are redundant [9,10]. In theory, this limitation could be addressed by augmenting the training data with millions of AlphaFold2 predicted structures: doing so can improve performance [11]. However, large-scale structure prediction and training on millions of structures is computationally intractable for most groups, and while AlphaFold2 predictions are generally of good quality, they are not perfect and may introduce bias.

Sequence and structure encoders each have fundamental but complementary limitations, both in terms of data availability and the richness of the modality. We thus ask the following question:

How can we leverage the availability of protein sequences and the spatial information in protein structures to jointly improve both sequence and structure encoders?

Several groups have developed models that combine sequence and structure information. Knowledge-Design and LM-Design combine learned structure encoders with ESM sequence encoders to improve protein sequence design [12,13]. RFDiffusion, a state-of-the-art diffusion generative model that operates in structure space, is fine-tuned from RoseTTAFold, a structure prediction model that was trained with hundreds of thousands of sequences [5,14]. ProstT5 uses a language model to embed both the protein sequence and a one-dimensional string representation of the protein structure [15,16]. The resulting embeddings can be used to predict protein properties and redesign sequences compatible with a fixed backbone structure [15]. These models demonstrate the power of using both sequence and structure information in deep learning methods.

### Contributions

We propose a new self-supervised objective, namely residue level alignment (RLA), to align the sequence and structure embedding spaces. Specifically, starting with a pretrained sequence encoder (a large language model such as ESM-2) and a randomly initialized structure encoder (a message-passing neural network, or MPNN), we encourage the latent representation of each residue in the structure embedding to match that residue’s representation in the sequence embedding (and vice versa). By aligning these two spaces, RLA enriches the sequence encoder with high-resolution structural information and enables the structure encoder to take advantage of large sequence databases. We demonstrate that RLA does the following:

#### (i) Injects spatial information into the sequence encoder.

RLA enhances the sequence encoder by indirectly providing structural supervision. RLA improves the performance of ESM-2 on unsupervised contact predictions and stability energy predictions.

#### (ii) Identifies complementary sequences and structures.

Using RLA, we can rank structural decoys according to their similarity to a corresponding sequence at a comparable level of performance to AlphaFold2, at a fraction of the computational cost [17].

#### (iii) Facilitates binder design.

Using RLA, we demonstrate that we can screen for appropriate docking interactions better and faster than AlphaFold2 Initial Guess, the current state of the art, even when the sequence for a candidate binder is not specified [18].

The success of RLA at these tasks demonstrates that synthesizing sequence and structure information by aligning their respective embedding spaces produces a richer computational representation of a protein than using sequence information or structure information alone.

## II. METHODS

Our aim is to design two encoders—a sequence encoder and a structure encoder—each of which accepts its own modality but whose latent space is informed by the other modality. Thus, our sequence encoder takes advantage of the spatial information present in structure data without requiring the actual protein structure (which is not known for most sequences), and our structure encoder takes advantage of large sequence databases without needing a specific protein sequence (which is not present in many design tasks).

At least two other groups have applied contrastive learning in the protein space. Palepu *et al*. [19] and Bhat *et al*. [20] developed PepPrCLIP, a contrastively learned model that aligns the ESM-2 embeddings for the sequences of a protein and its peptide binding partner and uses the aligned score to design new binders. Wang *et al*. [21] developed S-PLM, which aligns the ESM-2 embeddings of a sequence with the ResNet50 embeddings of its contact map, and showed that the resulting sequence embeddings better predict structural features. To our knowledge, RLA is the first method that applies contrastive learning at the residue level to align sequence and structure embeddings.

**FIG. 1.**
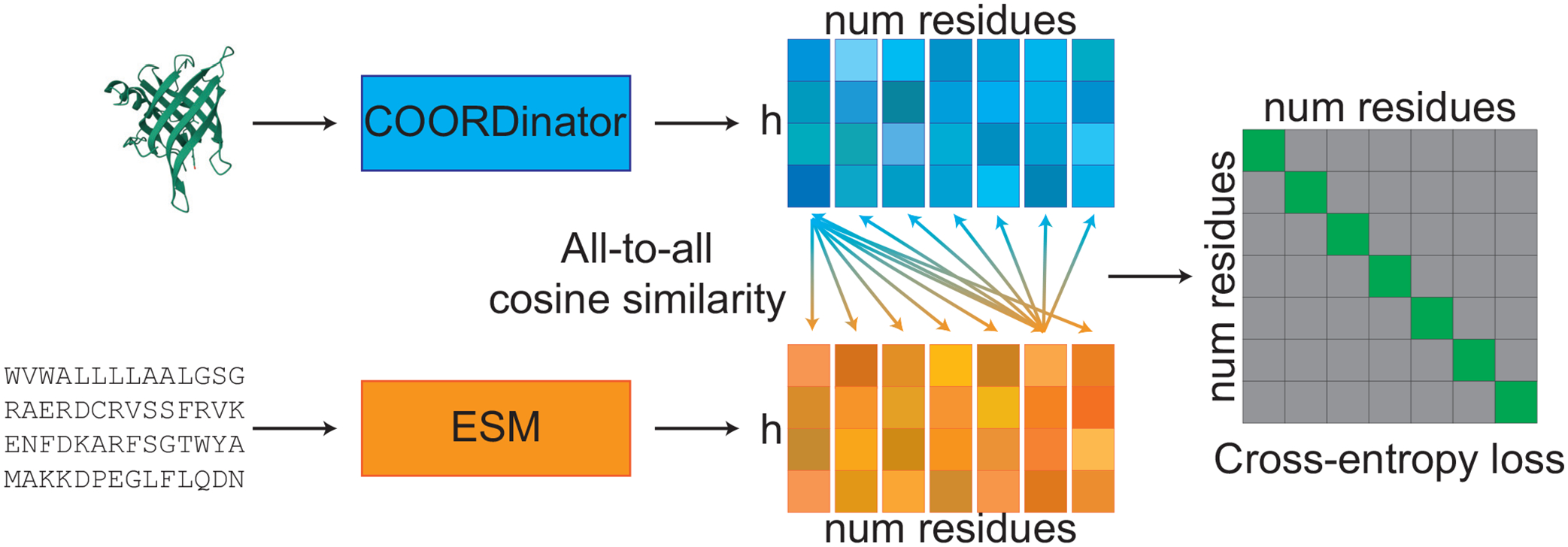
RLA training minimizes the cross-entropy loss for corresponding structure (blue) and sequence (orange) embeddings of dimension h for all num residues in a protein.

The key idea underlying RLA is to leverage the fact that protein sequences and structures share the same underlying subunit: the residues, i.e., the linked amino acids. Each residue in the protein sequence has an associated position in the protein structure. Our encoders output a sequence and structure embedding for each residue, and our training objective is to make the structure embedding of a residue more aligned with its corresponding sequence embedding than with the sequence embeddings of other residues in the same protein and vice versa (Fig. 1). Note that, unlike traditional cross-modal contrastive learning approaches (e.g., CLIP [22]), this residue level approach does not require a large batch size, making it much easier to scale.

### A. Residue level alignment

Given a protein with T residues, let $\left\{U_{T}\right\},\left\{V_{T}\right\}\in R^{d}$ be the sequence and structure embeddings for each of the residues. We use d=320. We define the RLA alignment score for residues i,j as the cosine similarity $r_{\text{RLA}}\left(U_{i},V_{j}\right)=\frac{U_{i}^{T}V_{j}}{\left\|U_{i}\right\|\left\|V_{j}\right\|}$ between the sequence embedding for i and structure embedding for j. We supervise a cross-entropy loss to maximize $r_{\text{RLA}}$ for a sequence/structure pair for a single residue relative to the $r_{\text{RLA}}$ between different residues

$${ℒ}_{\text{RLA}}=\frac{1}{T}\sum_{i=1}^{T}\text{log}\;\frac{r_{\text{RLA}}\left(U_{i},V_{i}\right)}{\sum_{j=1}^{T}r_{\text{RLA}}\left(U_{i},V_{j}\right)}+\frac{1}{T}\sum_{j=1}^{T}\text{log}\;\frac{r_{\text{RLA}}\left(U_{j},V_{j}\right)}{\sum_{i=1}^{T}r_{\text{RLA}}\left(U_{i},V_{j}\right)}.$$

### B. Positional information

Positional information can cause the model to “cheat,” for example by always giving the first residue the same embedding regardless of the input protein. To avoid this, we remove any positional information from the structure encoder, and we shuffle the order of the chains in a protein complex before training to ensure that the sequence and structure encoders do not see the chains in the same order.

### C. RLA similarity score

We compute a similarity score to compare a candidate sequence and structure. For chains 𝒞 and sequence and structure embeddings $\tilde{U}=\left\{{\tilde{U}}_{c}|c\in 𝒞\right\}$ and $\tilde{V}=\left\{{\tilde{V}}_{c}|c\in 𝒞\right\}$, each with $T_{c}$ residues, we define the similarity score by averaging the RLA scores over all residues per chain, $S_{\text{RLA}}(\tilde{U},\tilde{V})=\frac{1}{\left\|𝒞\right\|}\sum_{c\in 𝒞}\;\frac{1}{T_{c}}\sum_{i=1}^{T_{c}}\;r_{\text{RLA}}\left({\tilde{U}}_{c,i},{\tilde{V}}_{c,i}\right)$. For docking applications, we only average the RLA scores corresponding to the residues in each chain at the binding interface. The binding interface is defined as all residues that have a residue from the opposing chain in their 30 nearest neighbors.

We leverage a pretrained ESM-2 sequence encoder and a randomly initialized COORDinator structure encoder (see Sec. II D). We then perform RLA training on the structures within the PDB [9,10] (see Secs. II E and II F for hyperparameters and data set splits).

### D. Single modality encoders

For our sequence encoder, we fine-tune all layers of a pretrained ESM-2 model (150M parameters) [6,7]. This language model is trained on over 60 million protein sequences with an objective of recovering the identity of masked tokens. ESM-2 residue embeddings reflect biochemical properties and evolutionary conservation [23]. The attention weights derived from ESM-2 sequence embeddings can be used to predict contact maps, and the embeddings themselves form the basis for the structure predictions of ESMFold [6,7]. However, without structural information, ESM-2 contact maps are vulnerable to false positives [6,7] and, without fine-tuning, can be insensitive to single amino-acid substitutions [23].

For our structure encoder, we use COORDinator, an MPNN designed to predict sequences that are compatible with an input protein backbone structure [3]. COORDinator operates on a k-NN backbone structure graph with residues as nodes and interactions between residues as edges. Following other MPNN-based sequence predictors, we use k=30 [24]. Node features are initialized as an encoding of the three local dihedral angles for each residue, and edge features are initialized as an encoding of the combination of the relative residue positions and orientations and of all pairwise backbone atom distances for each pair of interacting residues [4,24]. Node and edge features are updated by alternating edge-update and node-update message-passing layers. For an edge, the update is computed based on the current edge feature and the current features of the nodes that the edge connects; for a node, the update is computed based on the current node features of all k neighbors and the updated features of the edges that connect the node to its neighbors [3]. We use randomly initialized weights instead of pretrained COORDinator weights to bias the resulting joint embedding space towards the ESM-2 embeddings.

### E. RLA hyperparameters

We implement our model using PyTorch [25] and train with the hyperparameters shown in Table I.

### F. Training data

We train on the PDB. Following the procedure from AlphaFold2 and ESMFold, we split the PDB examples based on a temporal cutoff: examples added to the PDB before 2021-08-01 are put into the train split, and those added after are randomly divided into the validation and test splits. We exclude from the train set the proteins in the data sets used in downstream inference tasks (see Sec. II G). This results in ~170 000 training proteins, ~15 000 validation proteins, and ~15 000 test proteins.

### G. Inference data

#### a. Mutation data.

To evaluate predicting the effect of sequence mutations on protein stability, we use the Megascale data set from Tsuboyama *et al*. [26]. This data set is comprised of cDNA display proteolysis thermal stability data for 500 natural and *de novo* designed small protein domains. For each protein, thousands of single and double mutations were made. There are a total of 776 000 measurements.

#### b. Monomer decoy data.

To evaluate ranking structural decoys, we use structure predictions made during the 2020 Critical Assessment of Structure Prediction (CASP) competition [27,28] compiled by Roney and Ovchinnikov [17]. The data set is comprised of the top 150 predictions for 81 CASP targets.

#### c. Protein-protein binding data.

To evaluate ranking protein-protein complexes, we use docking predictions made during Critical Assessment of Predicted Interactions (CAPRI) competitions compiled by Lensink and Wodak [29]. The data set is comprised of thousands of predictions for 10 target complexes.

#### d. Protein-peptide binding data.

Peptides are amino acid chains of fewer than 50 residues that are often unfolded when not bound to a target protein. To evaluate ranking protein-peptide complexes, we generated a data set using CABS-dock, a docking method [30]. We identified 27 protein-peptide interaction structures and used CABS-dock to generate 10 decoy structures for each.

#### e. Protein-miniprotein binding data.

Miniproteins are small proteins with a generally stable structure. To evaluate scoring experimentally validated protein-miniprotein complexes, we use miniproteins designed to bind to 10 protein targets generated by Cao *et al*. [31]. The data set is comprised of binding affinity measurements for tens of thousands of designs per target. Following Bennett *et al*. [18], we use a cutoff of $k_{D}⩽4\mu \text{M}$ to define binders. We filtered out two protein targets (TrkA and Tie2) with 10 or fewer successful binders and two other protein targets (H3 and EGFR) that no method performed well on. Statistics for the remaining targets are shown in Table II.

To analyze the variability in binding site location, given a set of coordinates ${ℛ}_{𝒯}$ for the Cα atoms of a target 𝒯 with n binders and a set of coordinates ${ℛ}_{{ℬ}_{i}}$ for the Cα atoms of binder ${ℬ}_{i}$, we define the binding site as ${𝒮}_{{ℬ}_{i}}=\left\{r|r\in {ℛ}_{𝒯}\right.,\left.{\text{min}}_{b\in {ℛ}_{{ℬ}_{i}}}(\|r-b\|)<8\;Å\right\}$. That is, the binding site is all residues on the target within 8Å of the binder. For two binding sites ${𝒮}_{{ℬ}_{i}}$ and ${𝒮}_{{ℬ}_{j}}$, the binding site overlap is defined as $L_{i,j}=\frac{\left\|{𝒮}_{{ℬ}_{i}}\cap {𝒮}_{{ℬ}_{j}}\right\|}{\left\|{𝒮}_{{ℬ}_{i}}\cup {𝒮}_{{ℬ}_{j}}\right\|}$. The average binding site overlap O for target 𝒯 is defined as $O_{𝒯}=\frac{2}{n^{*}(n-1)}\sum_{i,j>i}^{n}\;L_{i,j}$.

To cluster the binders, we calculate the structural similarity of ${ℬ}_{i}$ and ${ℬ}_{j}$ using the TM-score (denoted $T_{i,j}$), which ranges from 0 to 1, with 0 representing no similarity and 1 representing structural identity [32]. Each miniprotein binder comes from a scaffold, so we only cluster binders with a shared scaffold. We take a greedy approach to perform the clustering within a scaffold class. First, we define 𝒟 as the set of clustered binders, which starts empty. Second, we calculate $T_{0,j}$ for all j and form cluster ${𝒞}_{0}=\left\{j|T_{0,j}>0.8\right\}$. This represents the cluster of all binders similar to binder ${ℬ}_{0}$. Third, for a given binder ${ℬ}_{i}$, if i∈D, we assume it is already in the correct cluster and proceed to binder ${ℬ}_{i+1}$. If i∉D, we calculate $T_{i,j}$ for all j such that j∉D and ${\text{min}}_{k<i}\left(\left\|T_{i,k}-T_{j,k}\right\|\right)<0.25$. That is, we compute the TM-score between ${ℬ}_{i}$ and ${ℬ}_{j}$ if and only if j is not in a cluster and there does not exist another binder ${ℬ}_{k}$ such that ${ℬ}_{i}$ and ${ℬ}_{j}$ differ from ${ℬ}_{k}$ in different ways. Then, we form cluster ${𝒞}_{i}=\left\{j|T_{i,j}>0.8\right\}$ and update $D:D=D\cup {𝒞}_{i}$. Fourth, we repeat step three until all binders have been clustered. Once we have the clusters, we assign binder labels to each cluster based on whether the success rate in the cluster is higher than the overall success rate in the scaffold class to which the cluster belongs.

### H. Contact prediction

We follow the same protocol used for unsupervised contact prediction as described by Bhattacharya *et al*. [33] and Rao *et al*. [34]. Two residues are defined to be in contact in a structure if their Cα carbons are within 8Å of each other. Contacts between residues i and j are separated into short (6<|i-j|⩽12), medium (12<|i-j|⩽24), and long contacts (|i-j|>24) based on their separation in the sequence. The attention heads (with average product correction [34]) were extracted from the sequence encoder, and a linear probe was trained to classify the residue contacts. We trained the linear probe using a structurally split subset of our full data set: i.e., structures in the test set for this task are not similar to any structures in the train set [3]. There were 100 proteins in the train set, 20 proteins in the validation set, and 100 proteins in the test set. We assessed the structure and sequence similarity between proteins in this test set and proteins in the full train set and did not observe any bias in the performance of RLA-ESM (Fig. S1) [35].

### I. Test-train overlap

We used Foldseek to assess the sequence and structural overlap of any proteins used in testing to the proteins in the train set [16]. For monomer test proteins, we used the easy-search command, and for multimer test proteins, we used the easy-complexsearch command. For complexes that Foldseek reported were similar, we used TM-align to assess the similarity of the binding sites of the two complexes [36].

## III. RESULTS

### A. RLA injects spatial information into the sequence encoder

As a result of aligning the sequence and structure embeddings, fine-tuning ESM-2 with RLA makes the sequence model more structurally aware. We compare the performance of ESM-2 and RLA-ESM embeddings on predicting contacts between residues in a protein structure.

Rao *et al*. [34] found that the attention maps of ESM-2 contain structural contact information, i.e., whether two residues are in contact with each other in the folded protein structure. Specifically, they found that training a linear probe on top of the attention maps to predict residue contacts can compete with state-of-the-art contact prediction methods. However, ESM-2 contact prediction suffers from a low TNR. In Table III, we show that using RLA-ESM improves unsupervised contact prediction over short-, medium-, and long-range contacts. In particular, RLA-ESM reduces the number of false positives over all ranges.

Also, Meier *et al*. [37] demonstrated that ESM-2 embeddings can be used to predict the effect of mutations on protein energies. In Fig. S2 [35], we show that RLA-ESM does better than the baseline ESM-2 model at predicting protein stability energies [26].

### B. Exploring the joint RLA latent space

**FIG. 2.**
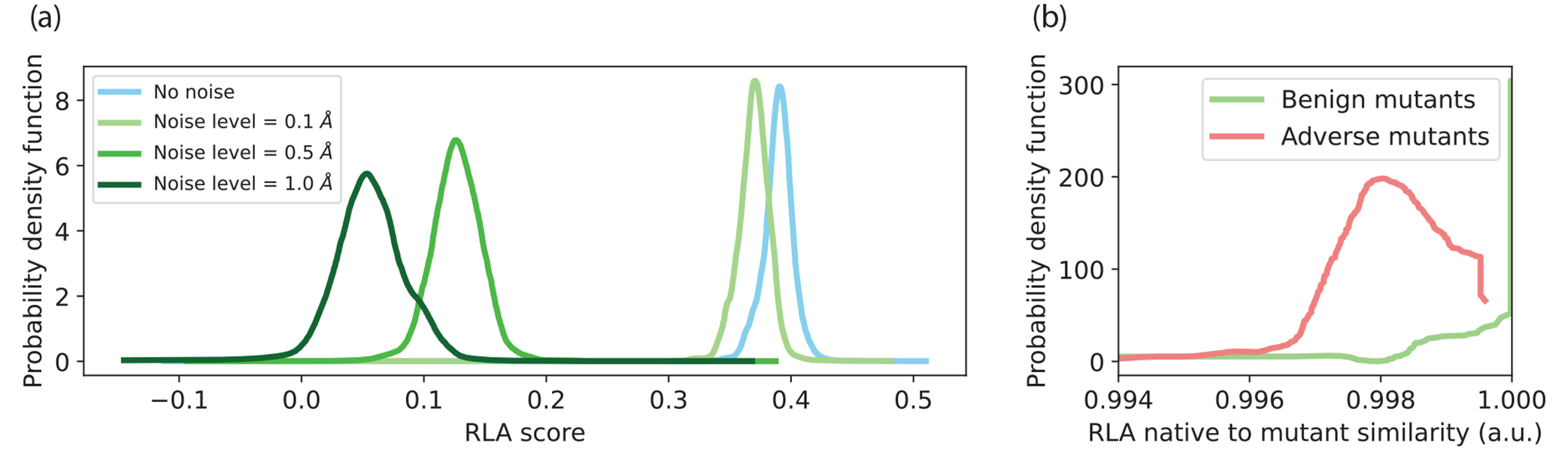
RLA scores behave as expected with simple structure and sequence perturbations. (a) RLA scores decrease as progressively more structural noise is added to the backbone coordinates of the encoded structure. Curves show the effects of adding Gaussian noise to give structural deviations of 0.1,0.5, or 1.0Å (light, medium, and dark green, respectively). (b) RLA-ESM embeddings are changed more by adverse mutations (red) than benign mutations (green) at protein-protein interfaces.

To explore the landscape of the joint latent space, we investigate how adding noise changes the alignment between sequence and structure pairs. As more noise is added to either the structure or the sequence, the representation of that modality should become progressively more misaligned. To test this, we take a random sample of proteins from our test set. As we add increasing amounts of Gaussian noise to the position of each atom in the protein structures, $r_{\text{RLA}}$ degrades [Fig. 2(a)]. Also, as we make mutations to all residues in the interface of 10 CAPRI protein-protein complexes [29], we observe that adverse mutations (negative BLOSUM62 scores) change the RLA score substantially more than benign mutations (positive BLOSUM62 scores) [38] [Fig. 2(b)]. These simple structure and sequence perturbations provide a baseline assessment of the feasibility of using the RLA latent space to measure sequence-structure compatibility.

### C. RLA identifies complementary sequences and structures

RLA positions both the sequence and structure embeddings within the same latent space. As a result, we can use RLA to identify complementary sequences and structures. In this section, we first evaluate RLA on a task that involves identifying native single-chain sequence-structure pairs in the presence of structural decoys. We then show how RLA can be used for binder design, i.e., to identify a new chain that stably binds to an existing chain, even when there is no sequence available for the designed binder.

#### 1. Using RLA to rank single-chain structural decoys

**FIG. 3.**
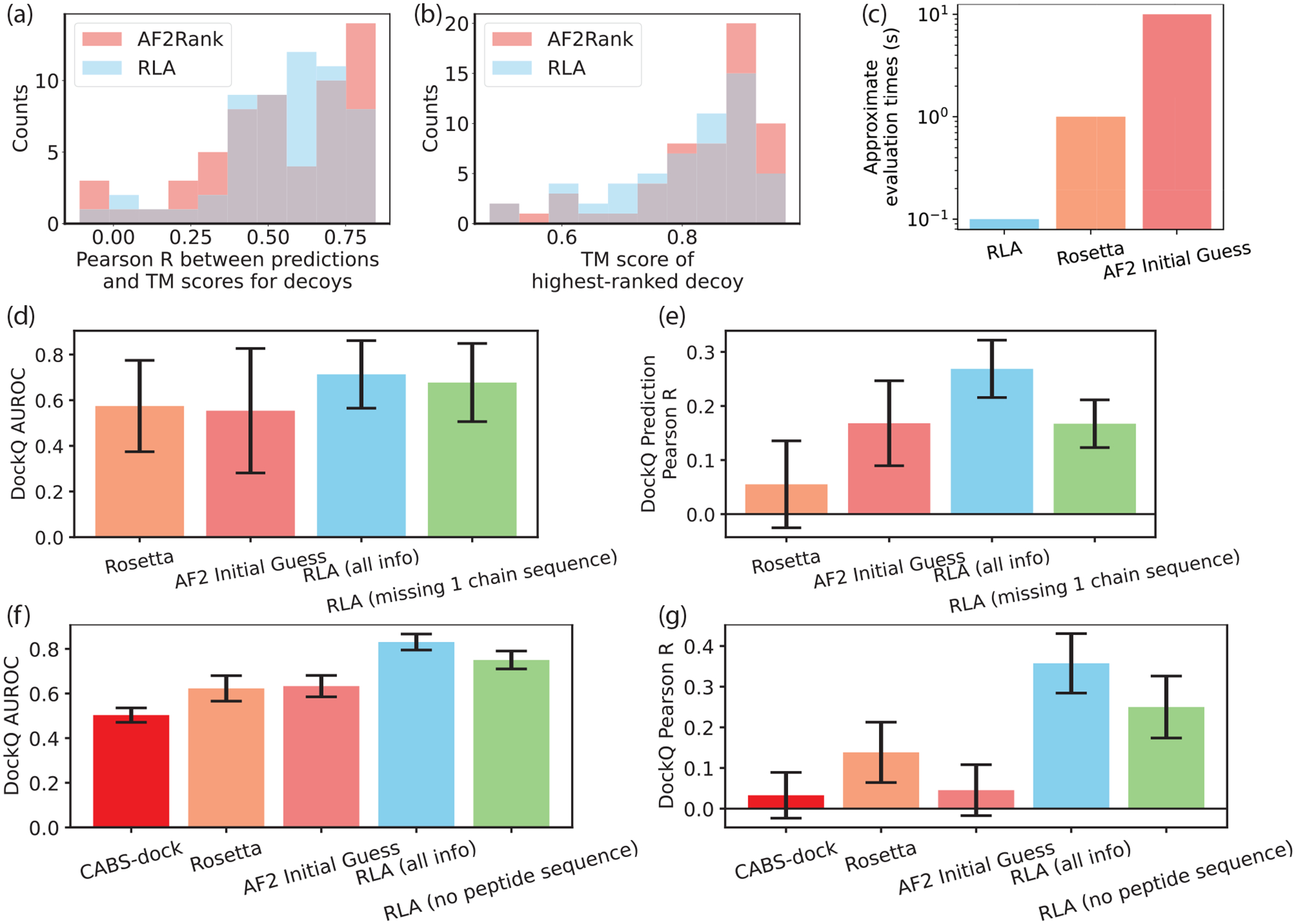
Performance discriminating between real and decoy structures. (a) RLA scores correlate with TM scores for single-chain structure predictions approximately as well as AlphaFold2 confidence scores (AF2Rank). (b) The highest-ranked decoy based on RLA score has a similar TM score to the highest-ranked decoy according to AF2Rank. (c) Approximate compute times per complex for each method used in the design tests. RLA scores discriminate between bad (DockQ<0.23) and good (DockQ>=0.23) decoy protein-protein (d) and protein-peptide (f) complexes better than Rosetta and AF2 Initial Guess, even without peptide sequence information. RLA scores correlate with decoy protein-protein (e) and protein-peptide (g) complex DockQ scores better than Rosetta and AF2 Initial Guess. Data shown are mean ± SEM over different targets.

Roney and Ovchinnikov [17] showed that AlphaFold2 can be used to rank structural decoys. In Figs. 3(a) and 3(b), we compare the efficacy of using AlphaFold2 versus RLA scores to discriminate between native and decoy sequence-structure pairs. The decoys were sourced from structure predictions during the 2020 CASP competition and vary in quality according to the template modeling (TM) score between the prediction and the solved structure. RLA scores are approximately as good as AlphaFold2 scores in predicting the quality of a decoy while requiring significantly fewer compute resources [Fig. 3(c)].

#### 2. Using RLA for binder design

Binder design is a longstanding problem where the task is to design the sequence and structure of a protein that binds a specified target. Here, we apply RLA to a subtask of binder design: discriminating good designs from bad ones. For the problem of *de novo* protein design, which operates outside of the sequence-structure space sampled by evolution, multiple sequence alignments (MSAs) are not available, and this limits the predictive power of AlphaFold2. For such design tasks, Bennett *et al*. [18] developed the AlphaFold2 Initial Guess (AF2 Initial Guess) model, which generates AlphaFold2-based scores for candidate design structures without the need for an MSA for the binder. We compare the performance of RLA and AF2 Initial Guess on binder discrimination.

#### 3. Evaluating binder decoy discrimination

As an initial test, we consider two data sets with decoy binder complexes: one with protein-protein interactions and the other with protein-peptide interactions. We evaluate how well different methods discriminate between acceptable and decoy complexes (according to DockQ scores [39]). DockQ scores range from 0 to 1, with a score above 0.23 denoting an acceptable docking pose. We evaluate performance by the AUROC for predicting acceptable complexes and by the correlation of the predicted score with the DockQ score.

When evaluating a candidate designed binder, both the protein sequence and structure are usually available. However, many design pipelines first create backbone structures and then design sequences for those that are most promising [40]. For such workflows, a decoy discriminator that is agnostic to the binder sequence is essential. Accordingly, we assess the performance of RLA using full protein sequences and compare this to the performance when the binder sequences are masked.

For the task of binder discrimination, we compare RLA scores with AF2 Initial Guess scores and Rosetta interface ΔΔG energies [41]. Rosetta energies were calculated using the InterfaceAnalyzer protocol with the following options: out:file:score_only, tracer_data_print, and add_regular_scores_to_scorefile. Both Rosetta and AF2 Initial Guess model the structure of side chains, which RLA does not. Side-chain structure information is often important to binder prediction models [42]. This should provide Rosetta and AF2 Initial Guess with an advantage in this task.

We first consider the CAPRI protein-protein interaction decoys. RLA scores perform substantially better than AF2 Initial Guess scores or Rosetta energies in differentiating acceptable and decoy complexes. Notably, this is true even when calculated without the sequence information for one of the chains (selected randomly) [Figs. 3(d) and 3(e)]. For CABS-dock protein-peptide interaction decoys, RLA scores are also better able to predict which decoy complexes are acceptable binders compared to AF2 Initial Guess scores, Rosetta energies, or CABS-dock scores [Figs. 3(f) and 3(g)]. Again, this is true even when calculated without the peptide sequence.

**FIG. 4.**
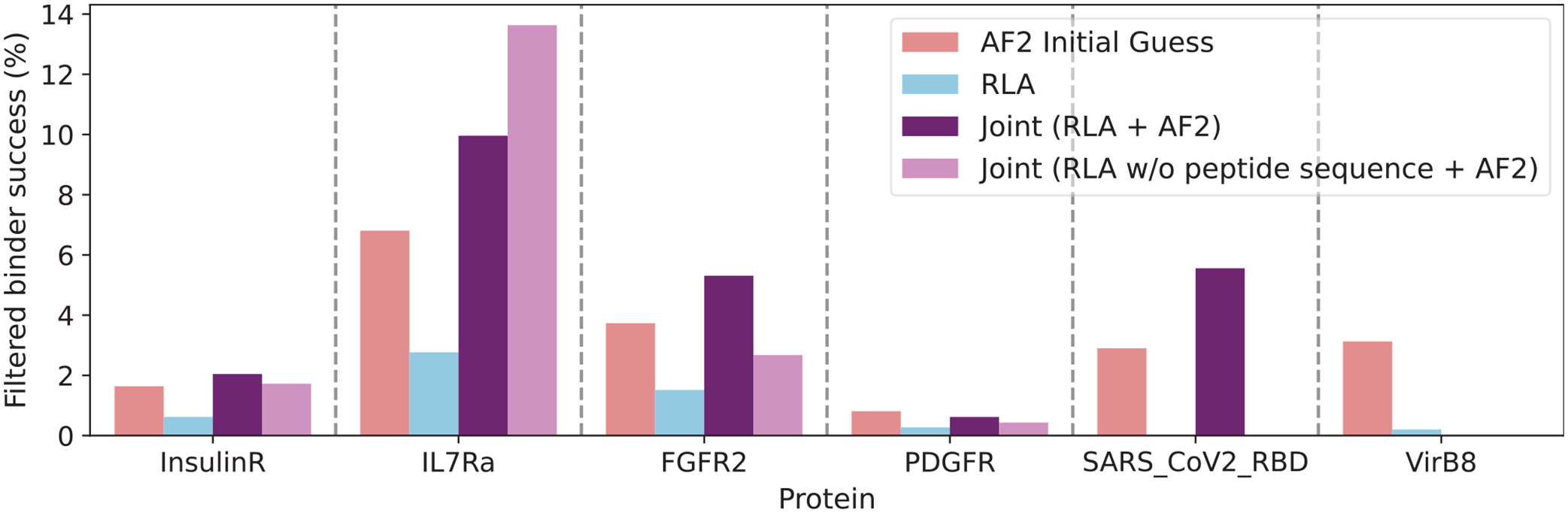
Binder success rates from filtering experimentally tested designs using AF2 Initial Guess, RLA, and the Joint Prediction (Joint) method.

In another test, we use the experimental binding data of Cao *et al*. [31] and assess how well different methods can detect successful miniprotein designs among many unsuccessful designs by calculating the percentage of filtered designs that are experimentally successful. We compare RLA with AF2 Initial Guess, using a cutoff of the top 25th percentile score as the RLA filter (a threshold empirically derived on these data) and pae_interaction < 10 as the AF2 Initial Guess filter, per Bennett *et al*. [18] (Fig. 4). RLA performs worse than AF2 Initial Guess on this task. Given that RLA scores are on average ~100 times faster to calculate than AF2 Initial Guess scores [Fig. 3(c)], we created a new Joint Prediction screening method that runs RLA on every binding candidate and then scores the top 25% with AF2 Initial Guess. This Joint Prediction approach performs better than either RLA or AlphaFold2 Initial Guess on almost all targets while reducing runtime by approximately 75%. Notably, the Joint Prediction method also works well when RLA scores are calculated without the binder sequence. We also calculate the false positive rate after filtering with each method, and using this metric AF2 Initial Guess performs the best (Fig. S3) [35].

There are two potential reasons why RLA underperforms on the miniprotein design benchmark (Fig. 4) compared to docking decoy discrimination (Fig. 3). First, this task involves selecting between miniproteins that were designed to engage binding sites that are substantially more similar to each other than are the binding sites of the protein or peptide binders in the decoy sets (Fig. S4a) [35]. Second, this task involves selecting between designed proteins with similar structures but different sequences, whereas docking decoy discrimination involves comparing different poses for the same protein partner with a single sequence. To test how sequence variation affects the performance of RLA and AF2 Initial Guess on detecting binders, we challenge the methods to identify the best binder structures from the Cao *et al*. [31] data regardless of the specific binder sequences (Fig. S4b) [35]. We cluster binders by structural similarity and label clusters as binders or nonbinders according to the binding success of their members (see Sec. II G). Each cluster has many different sequences but is assigned only one score: the RLA or AF2 Initial Guess score for a cluster is the mean of the scores for all cluster members (Figs. S4c,d and Fig. S5) [35]. The task of identifying successful clusters better reflects the ability of each method to discriminate binders based on their structures, making it more similar to the decoy discrimination tasks. RLA performs substantially better on the clustered data, matching the performance of AF2 Initial Guess (Fig. S4b) [35].

#### 4. Test-train overlap

In our Foldseek and TM-align tests for sequence, structure, and binding site similarity between proteins in the various test sets and proteins in the train set, there are many instances of similar proteins. This is expected, as we use a temporal cutoff for our data set split (and remove from the train and validation sets any proteins used in testing). For tasks that involve discriminating between the native structure and computational decoys, this could bias the performance of RLA. In Figs. S6–8 [35], we show that there is no relationship between RLA performance and sequence, structure, or binding site similarity between the test protein in question and the train set proteins. For the protein-miniprotein task, all of the structures being ranked were designed *de novo* and have very little sequence, structure, or binding site overlap with the train set (Fig. S9) [35].

## IV. DISCUSSION

Protein sequences and structures each support different types of analyses: while structures contain richer spatial context, sequence data are far more abundant. By aligning these two latent spaces together, RLA enables us to indirectly supervise each encoder with the other modality. We demonstrate that RLA enriches both the sequence and structure encoders compared to their single-modality counterparts. Specifically, we show that the RLA process improves the ability of the sequence encoder to predict structural contacts and to predict the effect of mutations on thermal stability. This demonstrates that by aligning the sequence and structural latent spaces, RLA provides indirect structural supervision to the sequence encoder, enabling it to represent a more accurate conception of the protein’s structure.

Moreover, by positioning both encoders in the same space, RLA can identify complementary sequences and structures, making it especially suitable for design tasks. We demonstrate that RLA outperforms AF2 Initial Guess, the state of the art for screening binding designs, on the task of identifying the correct protein-protein and protein-peptide docked complexes from sets of computationally predicted decoys. Also, RLA is approximately two orders of magnitude faster. On a data set containing experimental values for hundreds of thousands of miniprotein designs, RLA performs worse than AF2 Initial Guess. We show that the miniprotein design data set differs from the protein-protein and protein-peptide data sets in the variability of binding sites sampled and in the existence of binder sequence variation and that these differences partially explain the lower performance of RLA. Despite this, we demonstrate the power of using RLA in combination with AF2 Initial Guess through the Joint Prediction approach, which is the best performing screening method for the miniprotein designs.

Importantly, RLA can operate even when the binder sequence is not specified, a mode in which AF2 Initial Guess cannot operate. The Joint Prediction approach also remains very successful even when RLA is run without the binder sequence. These results and the computational efficiency of RLA relative to AF2 Initial Guess suggest that RLA is best applied early in the binder design process to prune large sets of structurally diverse designs without eliminating true positives.

The joint sequence-structure space opens up exciting avenues for future work. For example, in the computer vision space, conditioning on captions using a joint vision/language representation can improve image-to-text generation [43]. Drawing inspiration from this domain, RLA may provide a reliable way to condition protein diffusion models such as RFDiffusion. Given the success of RLA for ranking binder design candidates, this conditioning scheme might be particularly useful for generating peptides that bind to a provided protein sequence or structure.

Code for training RLA and calculating RLA scores, and data for benchmarks used, are available on GitHub [44].

## Supplementary Material

Supplement

## Figures and Tables

**TABLE I.** Hyperparameters for training RLA.

Name	Value
Batch size	10
Epochs	10
Scheduler	Cosine
Optimizer	Adam
Learning rate	0.001
Peak epoch	2
Weight decay	0.001

**TABLE II.** Statistics for the binder designs from Cao *et al*. [31].

Target	Number of designs	Number of successes	Success rate (%)
FGFR2	59 361	592	1.00
IL7Ra	14 921	22	0.15
InsulinR	58 772	244	0.42
PDGFR	99 211	283	0.29
SARS_CoV2_RBD	99 393	18	0.02
VirB8	9955	65	0.65

**TABLE III.** RLA fine-tuning improves unsupervised contact prediction maps derived by training a linear probe to predict residue contacts given language model attention maps. RLA gives improvements for short-, medium-, and long-range contacts, and in particular improves the true negative rate (TNR) by reducing the number of false positives for short- and medium-range contacts.

Distance	Method	Accuracy	TNR	TPR
*All*	ESM-2	0.816	0.905	0.727
	with RLA	**0.907**	**0.971**	**0.842**
*Short*	ESM-2	0.894	0.378	**0.953**
$\left|i-j\right|\in [6,12]$	with RLA	**0.935**	**0.790**	0.952
*Medium*	ESM-2	0.882	0.371	**0.955**
$\left|i-j\right|\in [12,24]$	with RLA	**0.937**	**0.839**	0.951
*Long*	ESM-2	0.796	0.932	0.591
$\left|i-j\right|\in [24,\infty ]$	with RLA	**0.898**	**0.979**	**0.776**
