# Supplementary material for: Jointly Embedding Protein Structures and Sequences through Residue Level Alignment
Source: PRX Life. Author manuscript; Available in PMC 2025 Oct 3. (PMC12490763; doi:10.1103/prxlife.2.043013)
Supplement: Supplement [file NIHMS2062694-supplement-Supplement.pdf]

Supplemental Materials for

Jointly Embedding Protein Structures and Sequences through Residue Level Alignment

Foster Birnbaum[1,2], Saachi Jain[3], Aleksander Madry[3], Amy E. Keating[1,4]

[1]Department of Biology, MIT, Cambridge, MA 02142
[2]Computational and Systems Biology Program, MIT, Cambridge, MA 02142
[3]Department of Electrical Engineering and Computer Science, MIT, Cambridge, MA 02142
[4]Department of Biological Engineering, MIT, Cambridge, MA 02142

Amy E. Keating
Address: 31 Ames Street, Cambridge, MA 02142
Phone: (617) 452-3398
E-mail: keating@mit.edu

Supplemental Figures

Figure S1. Contact map prediction test-train leakage………………………………………….. 2
Figure S2. RLA-ESM mutation effect prediction……………………………………………. 3
Figure S3. Miniprotein design filtering false positive rates…..………………………………. 4
Figure S4. RLA performance variation tests………..………………………………………. 5
Figure S5. Miniprotein cluster statistics………………..……………………………………. 6
Figure S6. CASP decoy discrimination test-train leakage…………...………………………. 7
Figure S7. Protein-peptide test-train leakage……………………….……………………... 8
Figure S8. Protein-protein test-train leakage…….………………………………….……….. 9
Figure S9. Protein-miniprotein test-train leakage ………………………………………….. 10

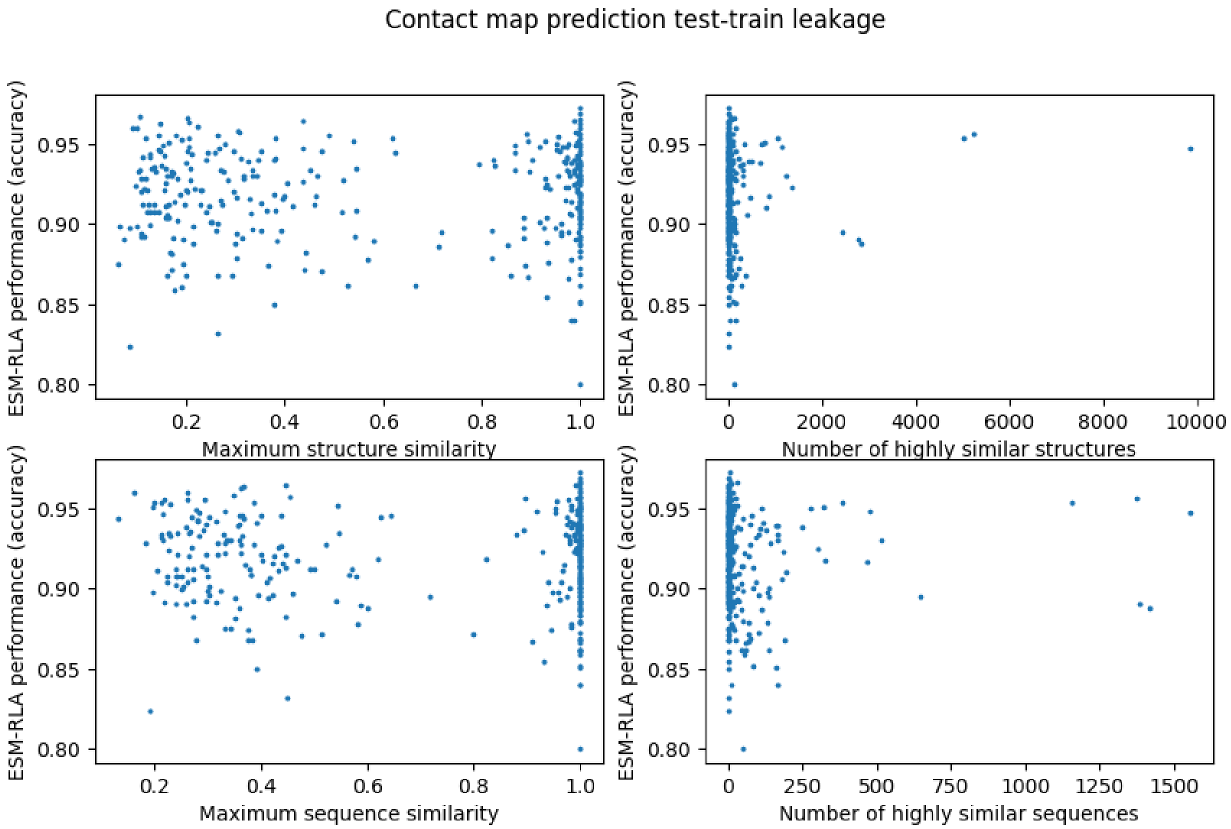

Fig. S1. There is no relationship between the performance of ESM-RLA on predicting contact maps and the sequence or structure similarity of proteins used in that task to proteins in the training set.

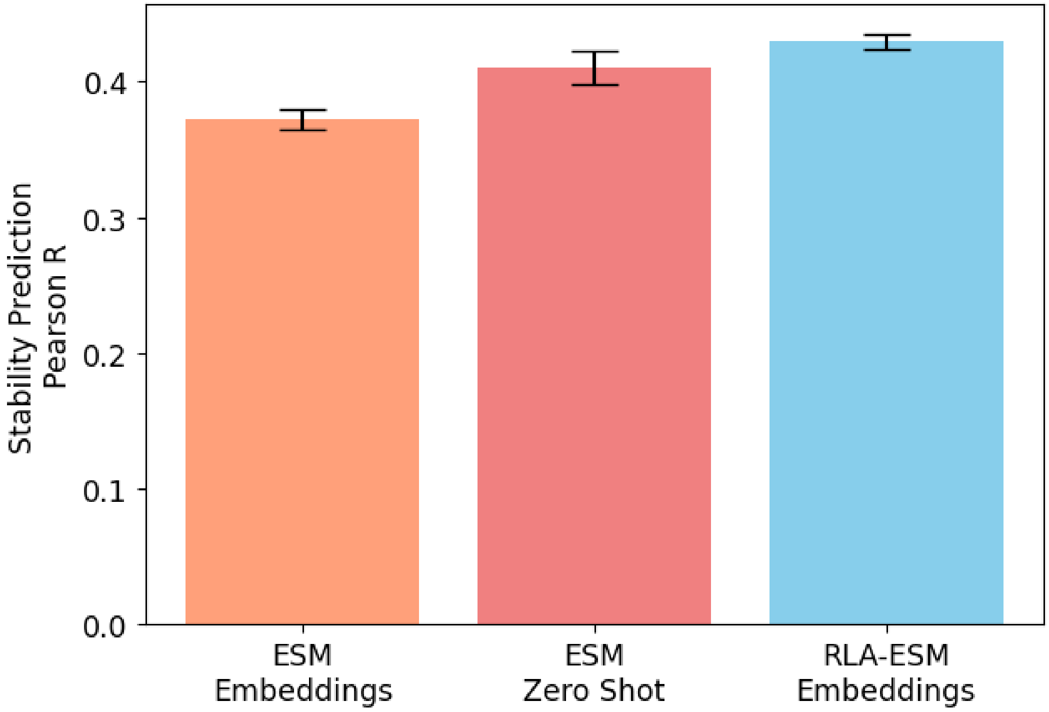

Fig. S2. RLA improves mutation effect predictions compared to using language model embeddings. ESM can be used to predict energies in two ways. First, ESM-2 embeddings can be used to predict the effect of mutations in a zero-shot fashion (ESM zero shot) using the language model head to compare the mutant and wild-type probabilities for a given residue. Second, the raw ESM-2 embeddings of the mutant and the wild-type protein can be directly compared (e.g., using cosine similarity) to score the impact of the mutation. Because we did not fine tune the language head to work with our RLA-ESM embeddings, we calculated mutation scores using the latter approach. RLA-ESM embeddings are better than baseline ESM embeddings or ESM zero shot for predicting protein stability energies. Data shown are mean ± SEM.

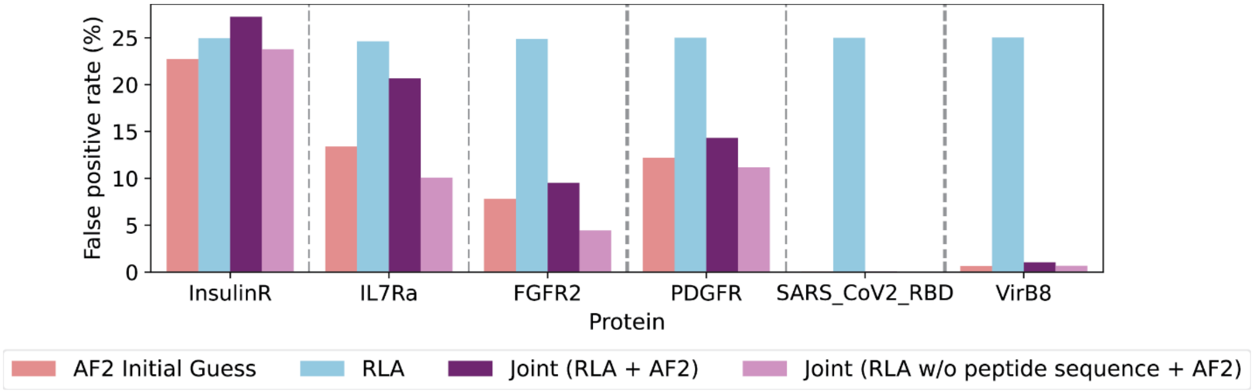

Fig. S3. Binder false positive rates from screening experimentally tested designs using AF2 Initial Guess, RLA, and the Joint Prediction (Joint) method. The data were generated using the same cutoffs that were applied to generate filtered binder success rates in Fig. 4.

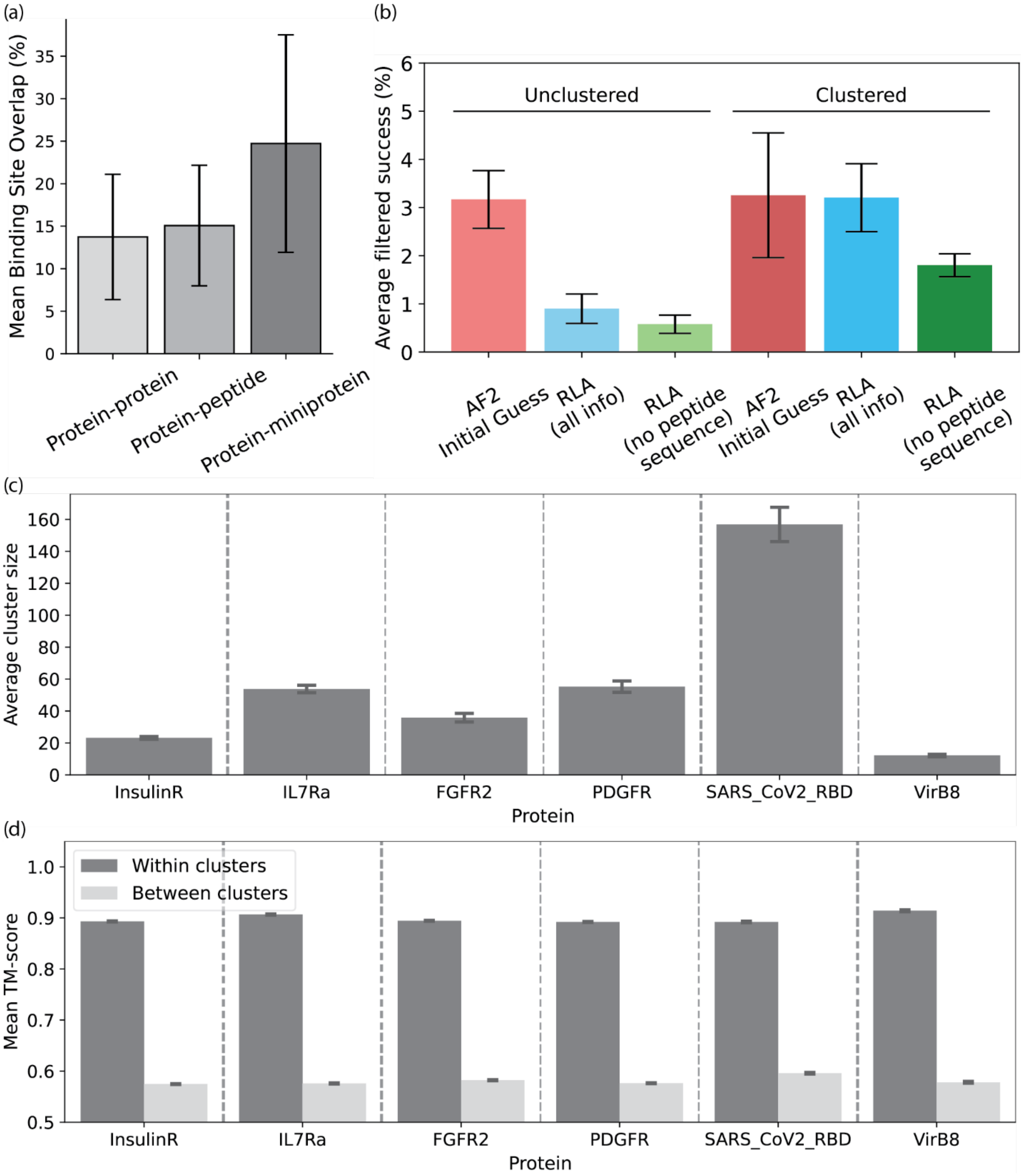

Fig. S4. Binding site similarity for different datasets and cluster-based test for filtering designed miniprotein structures. (a) Binding site similarity compared between the three binding datasets. (b) Filtered binder (unclustered) or cluster success rates using RLA and AF2 Initial Guess. (c) Average cluster sizes. (d) Average TM-score among proteins within clusters and between clusters. Data shown are mean ± SEM.

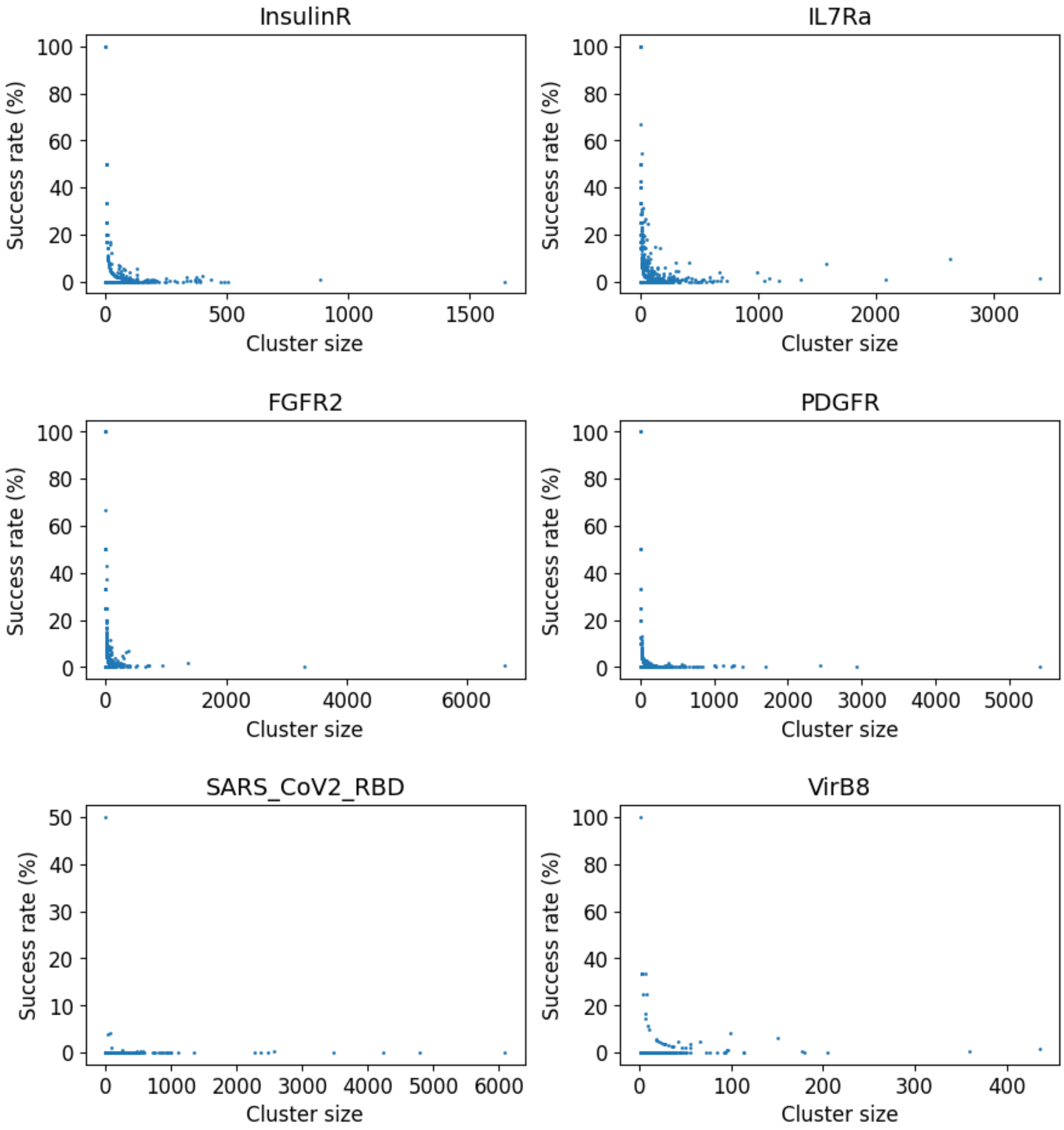

Fig. S5. Cluster sizes and the percentage of cluster members that are experimental successes for each target. Any cluster with a success rate greater than the overall success rate for the scaffold class to which the cluster belongs was designated as a binder cluster.

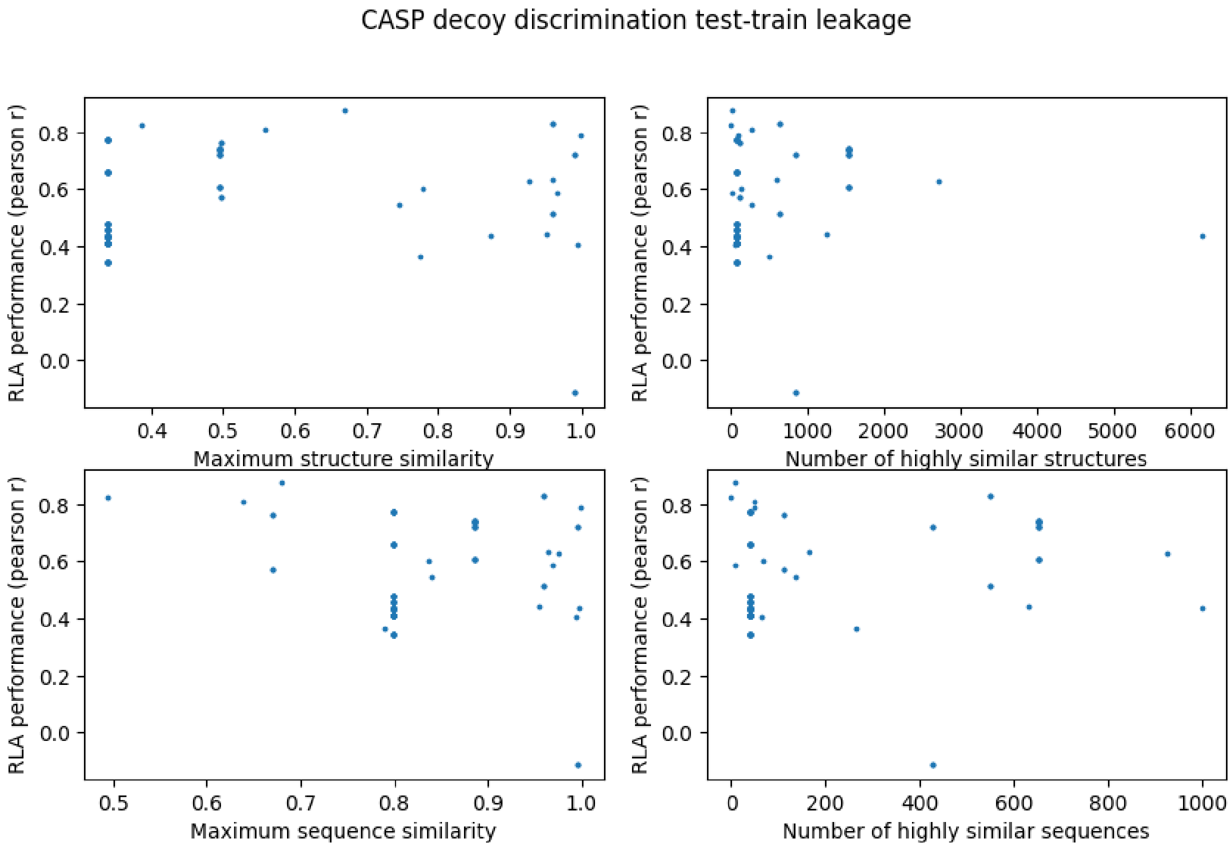

Fig. S6. There is no relationship between the performance of ESM-RLA on discriminating between good and bad decoys of CASP monomer proteins and the sequence or structure similarity of the CASP proteins to proteins in the training set.

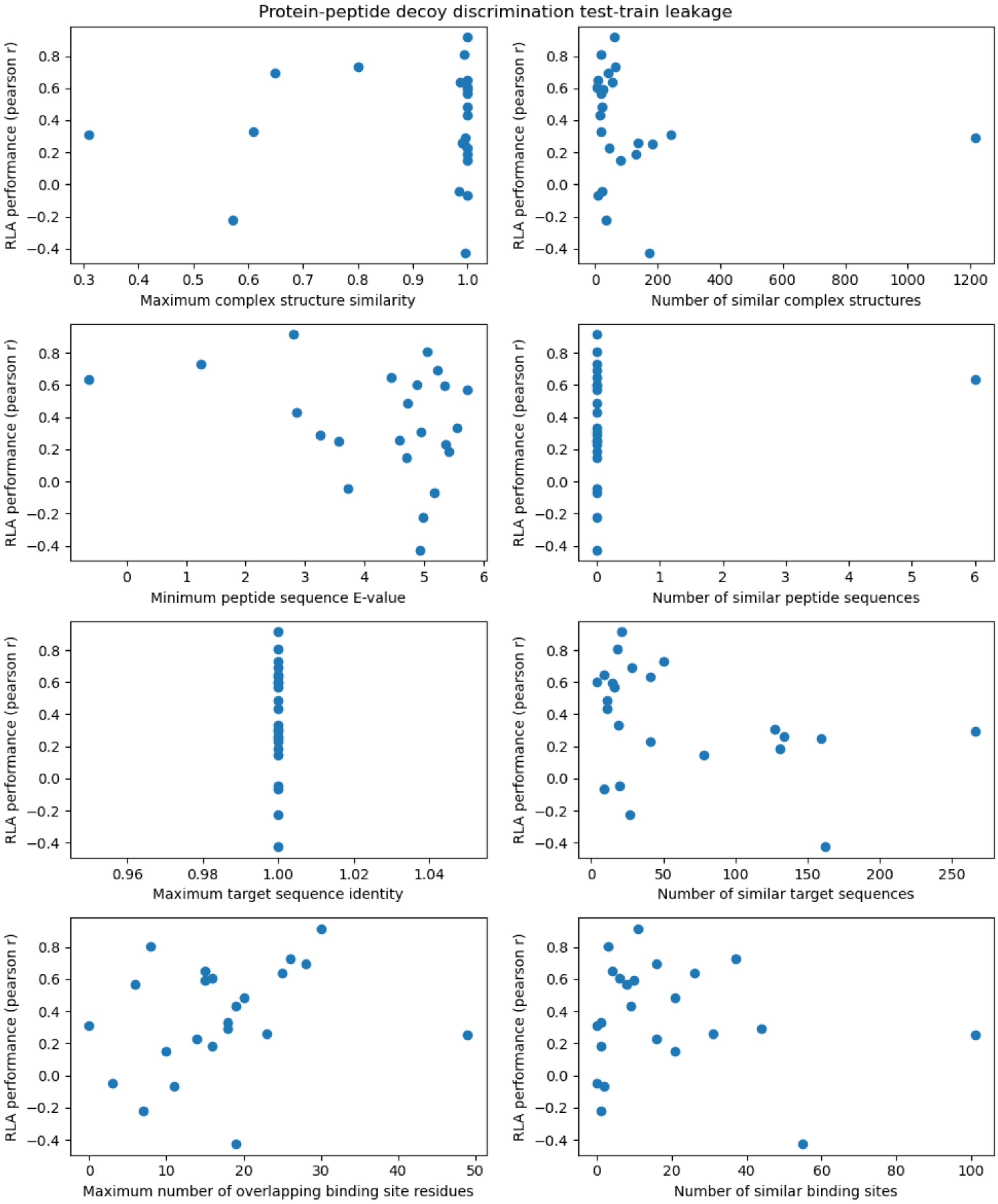

Fig. S7. There is no relationship between the performance of ESM-RLA on discriminating between good and bad decoys of protein-peptide complexes and the sequence or structure similarity of the protein-peptide dataset complexes to complexes in the training set.

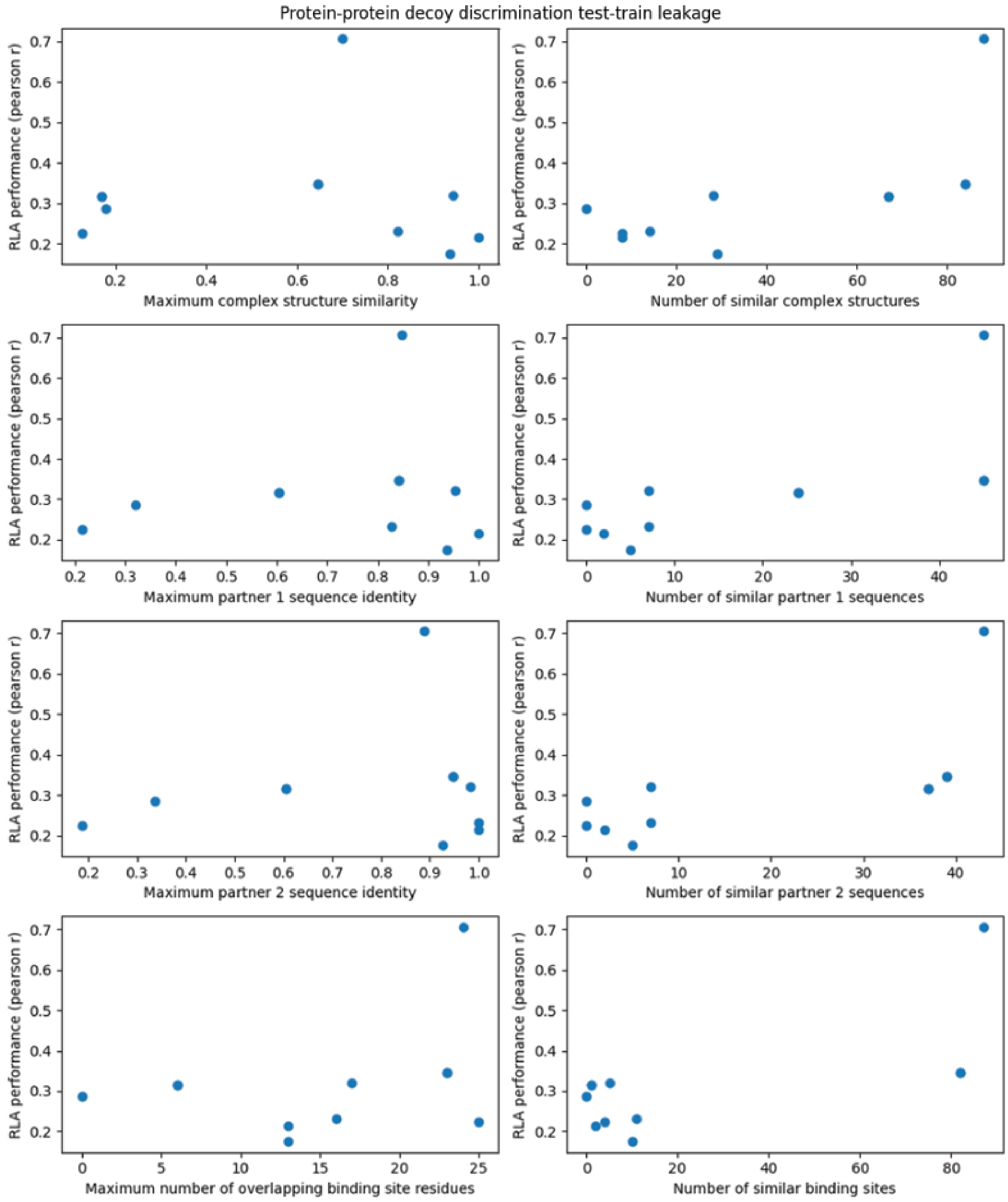

Fig. S8. There is no relationship between the performance of ESM-RLA on discriminating between good and bad decoys of protein-protein complexes and the sequence or structure similarity of the protein-protein dataset complexes to complexes in the training set.

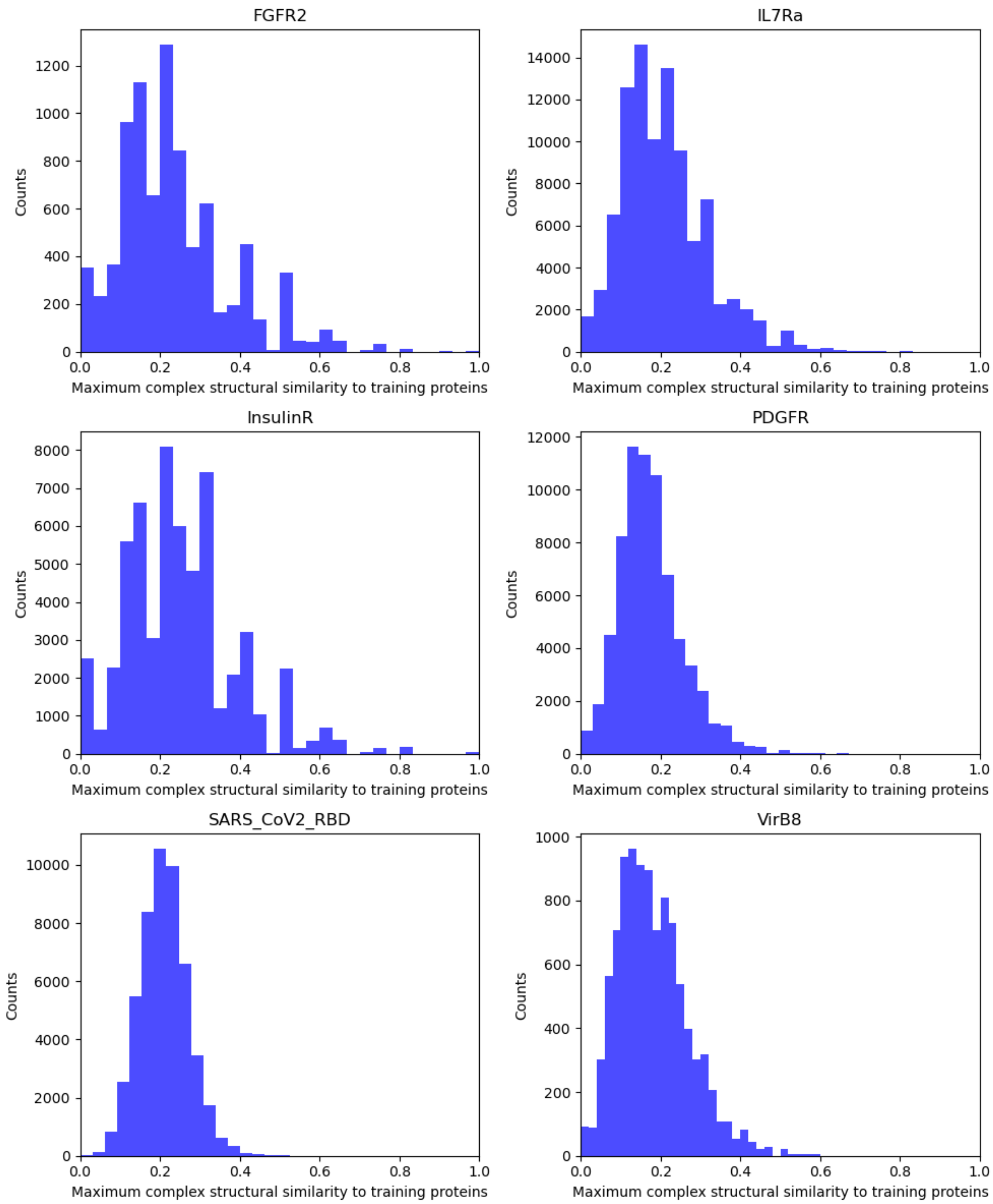

Fig. S9. The miniprotein designs tested in the protein-miniprotein binding task generally do not have high structure similarity to protein complexes in the training set.
